# A prognostic index model to individually predict clinical outcomes for colorectal cancer with synchronous bone metastasis

**DOI:** 10.7150/jca.40921

**Published:** 2020-05-11

**Authors:** Xu Guan, Chen-xi Ma, Ji-chuan Quan, Zhi-xun Zhao, Hai-peng Chen, Peng Sun, Song Wang, Zhao Lu, Xiao-long Ma, Zheng Liu, Zheng Jiang, Xi-shan Wang

**Affiliations:** 1 Department of Colorectal Surgery, National Cancer Center/National Clinical Research Center for Cancer/Cancer Hospital, Chinese Academy of Medical Sciences and Peking Union Medical College, Bejing, China; 2 Department of Colorectal Surgery, the Second Affiliated Hospital of Harbin Medical University, Harbin, China

**Keywords:** colorectal cancer, bone metastasis, nomogram, prognostic factors, cancer specific survival, the Surveillance, Epidemiology, and End Results database

## Abstract

**Background:** The prognosis of synchronous bone metastasis (BM) in colorectal cancer (CRC) is poor and rarely concerned. A clinical tool to evaluate the prognosis and clinical outcomes for BM would be attractive in current clinical practice.

**Methods:** A total of 342 CRC patients with synchronous BM were identified from Surveillance, Epidemiology, and End Results (SEER) database. The cancer specific survival (CSS) was estimated with the Kaplan-Meier method. Prognostic factors were identified from multivariate Cox model, and the final clinical nomogram was developed to predict the CSS. The concordance index (C-index) was used to assess the discriminative ability. Calibration curves were provided to internally validate the performance of the nomogram.

**Results:** The nomogram finally consisted of 6 prognostic factors including age, tumor grade, AJCC N stage, carcinoembryonic antigen (CEA) levels, primary tumor resection and chemotherapy, which translated the effects of prognostic factors into certain scores to predict the 1-, 2- and 3-year CSS for the synchronous BM in CRC patients. The nomogram presented a good accuracy for predicting the CSS with the C-index of 0.742. The calibration of the nomogram predictions was also accurate.

**Conclusions:** This nomogram was accurate enough to predict the CSS of CRC patients with synchronous BM using readily available clinicopathologic factors and could provide individualized clinical decisions for both physicians and patients.

## Introduction

Colorectal cancer (CRC) ranks the second most common cancer cause of death worldwide, leading to more than 881 thousand deaths in 2018^1^. Even bone metastasis (BM) is relatively rare in CRC ^2, 3^, the incidence of BM has gradually increased in recent years. BM is usually diagnosed at the advanced stages with a poor prognosis of 5-year survival rate with less than 5%^4^, and the skeletal-related events (SREs) such as bone pain, pathological fracture, possible radiotherapy, spinal cord compression and fatal hypercalcemia could further affect the survival of patients^5^. Therefore, the survival for each CRC patient with BM varies widely and there is no available evaluating system to provide prognosis prediction for this group of patients^6-8^.

Previous clinical reports regarding the treatment of CRC patients with BM mainly concentrated in studies with small sample size and limited follow-up duration^7, 9, 10^, which may lead to big bias for clinical practice. Furthermore, current treatment plan making for systemic and local control of CRC patients with BM were mostly originated from clinical experience because of the relative rarity of BM in CRC patients. Hence, the current therapy for BM clearly lacks strong evidence and rigorous guidance.

Nomogram is a reliable and alternative tool to quantify risk by incorporating and illustrating crucial factors for prognosis and it has been proved to make more precise survival prediction compared with traditional TNM staging systems^11-13^. In addition, nomogram could assist clinicians with making individual clinical decision making. Thus, the aim of our study was firstly to present the clinicopathological characteristics of CRC patients with BM from the nationwide population-based database, and then to develop a nomogram by utilizing readily available clinicopathologic factors, which could predict the prognosis and then potentially guide treatment decisions for CRC with synchronous BM.

## Materials and Methods

### Data resources

The patients with CRC diagnosed with synchronous BM were extracted from the Surveillance, Epidemiology, and End Results (SEER) database between January 2010 and December 2014. The SEER is an openly accessed database, which includes the information with regard to cancer incidence, survival outcome and treatment strategy from 17 population-based cancer registries and represents approximately 28 percent of the US population. Data in the SEER database do not require informed patient consent, because they were anonymized and de-identified prior to release. This study was approved by the Ethics Committee of National Cancer Center/National Clinical Research Center for Cancer/Cancer Hospital, Chinese Academy of Medical Sciences and Peking Union Medical College institutional review board.

### Study population

All CRC patients included in this study were definitively diagnosed by pathological examination, and BM were diagnosed using imaging examination and/or pathological examination. The cancer-specific survival (CSS) was defined as the time from the diagnosis until cancer-associated death and the end of follow up. Several clinical and tumor related variables were collected to analyze the prognostic impact on survival, including age, gender, primary tumor location, tumor grade, tumor size, pathological type, CEA levels, AJCC T stage, AJCC N stage, extra-osseous metastasis (involving bone, brain, liver and lung metastasis), primary tumor resection, radiotherapy and chemotherapy in SEER database.

### Statistical analysis

The CSS was assessed with Kaplan-Meier method, with the log-rank tests used to compare subgroups. In order to reduce the impact of sample size, potential prognostic factors with P<0.20 in univariate Kaplan-Meier analyses^14^ were finally entered into multivariate analysis via the Cox regression model. The nomogram was developed based on these prognostic factors (P<0.05) from the final (after forward selection) Cox model to predict the CSS of CRC patients with synchronous BM, and discriminative ability was appraised by concordance index (C-index). Calibration curves, which plot the average Kaplan-Meier estimate against the corresponding nomogram for 1-, 2-, or 3-year predicted CSS were provided to internally evaluate the performance of the nomogram based on the Cox model. In evaluating calibration, we stratified the patients into 3 equally sized subgroups and bootstrap-corrected CSS rates were calculated by averaging the Kaplan-Meier estimates based on 1000 bootstrap samples. All statistical analyses were performed with SPSS version 25.0 for Mac and R version 3.6.0. It is considered as statistically significant when P <0.05.

## Results

### Patient characteristics

**Figure 1:**
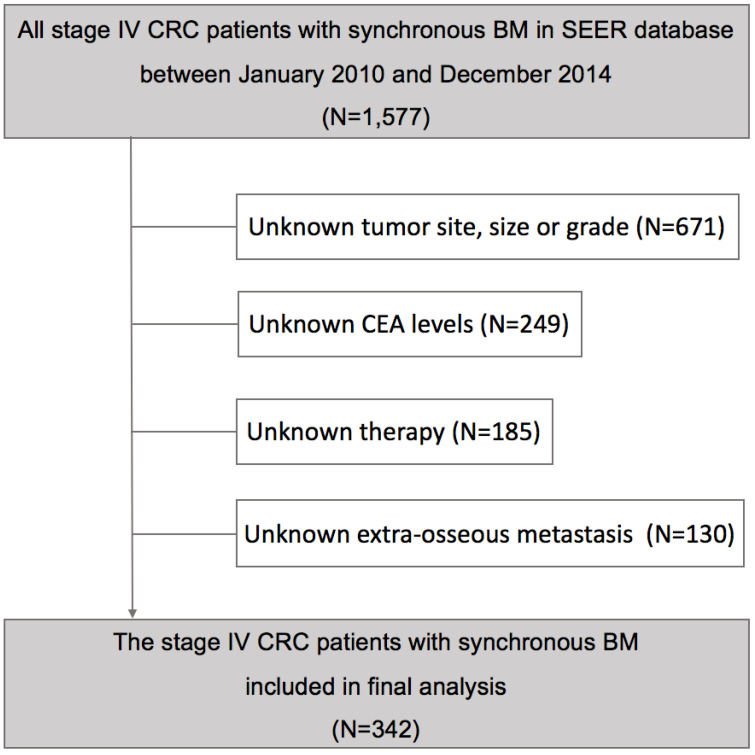
Analytical cohort and exclusion criteria of patients with synchronous bone metastasis from colorectal cancer.

After excluding 1,235 cases from SEER database who were not eligible, finally a total of 342 stage IV CRC patients with synchronous BM were conducted on our study **(Figure 1)**, whose clinical and tumor characteristics were shown in **Table 1**. Patients with age ≥60 (57.0%), colon cancer (66.4%), low tumor grade (63.1%), tumor size ≥5 cm (58.5%), adenocarcinoma (84.8%), CEA positive (81.3%), high AJCC T stage (85.4%) or lymph node metastasis positive (77.2%) had higher proportion. Extra-osseous metastasis was present in the majority of cases (78.4%), while the primary tumor was resected in 67.3% of cases. 116 (33.9%) cases received radiotherapy while 226 (66.1%) cases received chemotherapy in all patients.

### CSS analysis

**Figure 2:**
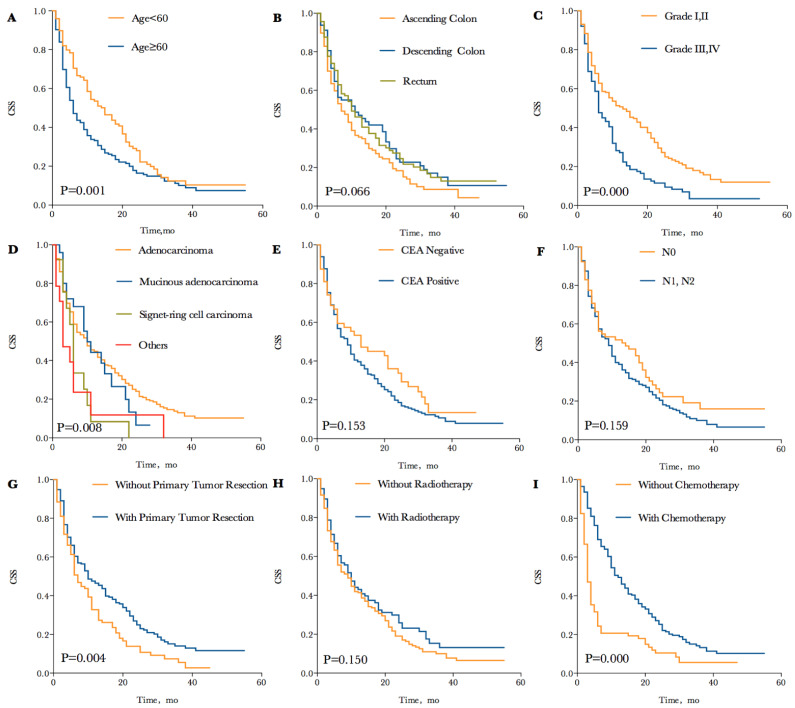
Kaplan-Meier cancer specific survival curves according to different potential variables with P<0.20. (A) Kaplan-Meier cancer specific survival curve according to age. (B) Kaplan-Meier cancer specific survival curve according to tumor site. (C) Kaplan-Meier cancer specific survival curve according to tumor grade. (D) Kaplan-Meier cancer specific survival curve according to pathological type. (E) Kaplan-Meier cancer specific survival curve according to CEA level. (F) Kaplan-Meier cancer specific survival curve according to N stage. (G) Kaplan-Meier cancer specific survival curve according to primary tumor resection. (H) Kaplan-Meier cancer specific survival curve according to radiotherapy. (I) Kaplan-Meier cancer specific survival curve according to chemotherapy.

There was a total of 257 (75.1%) patients died due to progressive CRC at the time of analysis. The median CSS in CRC patients with synchronous BM was 9 months, and the 1-, 2- and 3-year CSS of patients were 42.0%, 21.4% and 11.2%. Each survival curve of potential prognostic variable with P<0.20 in univariate Kaplan-Meier analyses were represented in **Figure 2**. These factors included age (P=0.001; median CSS= 15 and 6 months for age<60 and ≥60, respectively), primary tumor site (P=0.066; median CSS=7, 11 and 10 months for ascending colon, descending colon and rectum, respectively), tumor grade (P=0.000; median CSS=13 and 6 months for grade I, II and grade III, IV, respectively), pathological type (P=0.008; median CSS=10, 10, 6 and 3 months for adenocarcinoma, mucinous adenocarcinoma, signet-cell carcinoma and others, respectively), CEA levels (P=0.153; median CSS=13 and 9 months for CEA negative and positive, respectively), N stage (P=0.159; median CSS=14 and 9 months for N0 and N1,N2, respectively), primary tumor resection (P=0.004; median CSS=10 and 7 months for with and without resection, respectively) and radiotherapy (P=0.150; median CSS=10 and 9 months for with and without radiotherapy, respectively). Especially, CSS was remarkable longer for patients who received chemotherapy compared to those who did not (P=0.000; median CSS=14 and 3 months, respectively). The 1-, 2- and 3-year CSS of patients treated with chemotherapy were 54.0%, 27.8% and 14.6%, comparing to 18.2%, 8.7% and 4.6% of 1-, 2- and 3-year CSS without chemotherapy, which have striking differences in survival.

The final multivariable Cox model analysis of CSS was presented in **Table 2**. The significant prognostic variables including age (P=0.011), tumor grade (P=0.000), primary tumor resection (P=0.000) and chemotherapy (P=0.000) was identified in final Cox analysis. Moreover, the model also included CEA levels (P=0.005) and AJCC N stage (P=0.001) as the independent factors for CSS in CRC with synchronous BM, although neither were they significant in univariate Kaplan-Meier analyses (P=0.153 and P=0.159 respectively).

**Figure 3:**
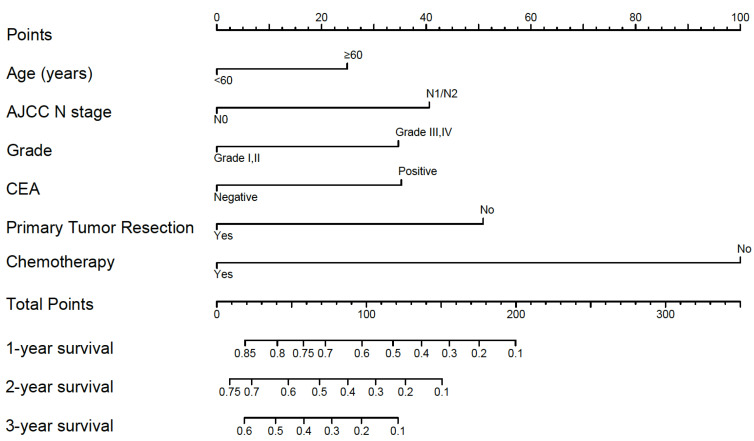
Nomogram for predicting 1-, 2- and 3-year cancer specific survival. Sum the points of each variable (age, AJCC N stage, grade, CEA, primary tumor resection and chemotherapy) and locate this sum on the Total Points axis. Then respectively draw the “1-year survival”, “2-year survival” and “3-year survival” axis to find the predicted cancer specific survival time.

**Figure 4:**
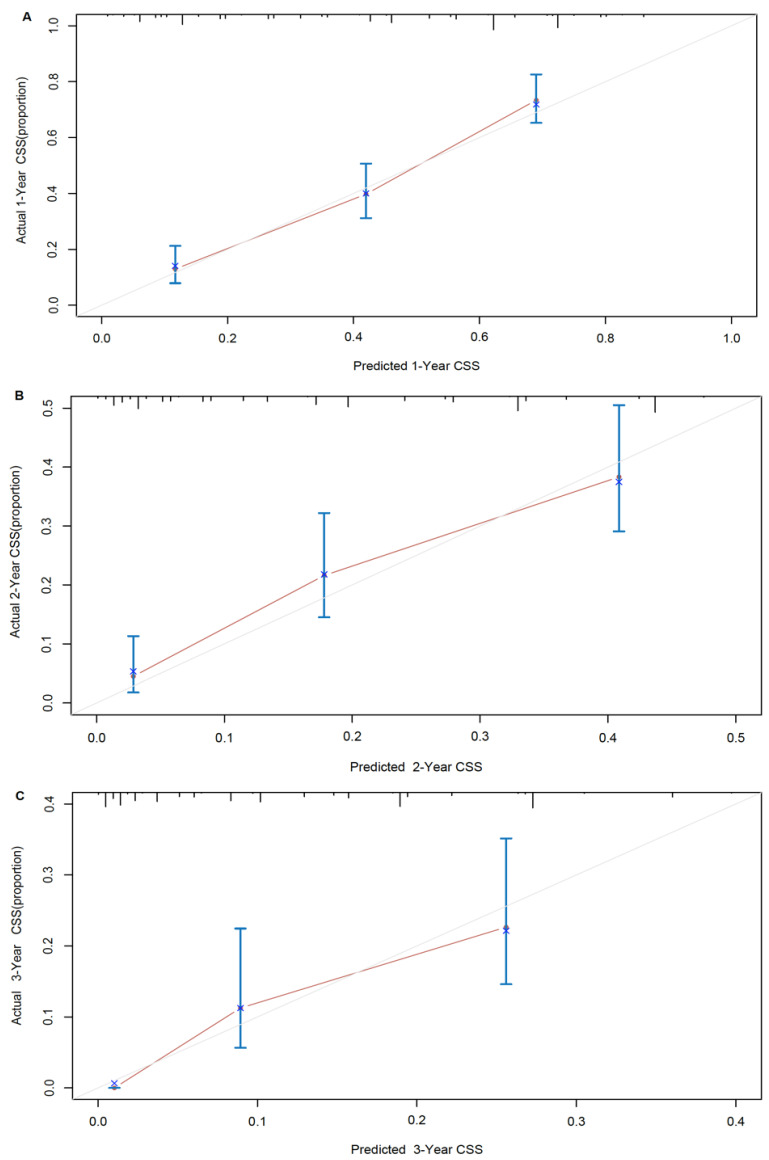
Nomogram model calibration curves of actual cancer specific survival with 95% confidence interval by decile (y-axis), over predicted cancer specific survival (x-axis) by nomogram: (A) 1-year nomogram calibration curve. (B) 2-year nomogram calibration curve. (C) 3-year nomogram calibration curve.

A nomogram developed by using the above six prognostic factors from the final multivariate Cox model was represented in **Figure 3**. The nomogram aimed to translate the effects of prognostic factors into certain scores and a weighted total score was calculated from these factors to predict the 1-, 2- and 3-year CSS for synchronous BM in CRC patients. Then we internally validated the nomogram which used the same set of data of 342 patients by bootstrap validation method, which presented good accuracy for predicting CSS of patients with a bootstrap corrected C-index of 0.742 (95% CI: 0.711-0.773). The calibration curves for 1-, 2- and 3-year CSS estimates showed good correlation between the CSS estimates from the nomogram and those derived from Kaplan-Meier estimates (**Figure 4**).

## Discussion

Effective prognostic assessment is crucial for CRC patients diagnosed with synchronous BM. However, there is no complete evaluating system to accurately estimate the prognosis of this group of patients for physicians^6-8^. The nomogram, a simple statistical predictive tool, is able to predict the survival and prognosis of BM more accurately for CRC patients with visualization results, which can further improve the compliance and therapeutic effect of patients. The SEER database provides potent data support for developing the nomogram.

Our study retrospectively analyzed the survival outcomes of total 342 CRC patients with synchronous BM. We finally identified variables including patients' age, tumor grade, CEA levels, AJCC N stage, primary tumor resection and chemotherapy as independent prognostic factors. Then we used these significant factors to develop nomogram. Because the nomogram only included six common clinicopathological variables, it could be used to accurately make precise prediction of survival and guide useful treatment. In addition, the C-index and internal validation also demonstrated this nomogram was a reliable tool for estimate of CSS in CRC patients with synchronous BM.

Here, we could find the prognosis of synchronous BM in CRC patients was very poor, which is consistent with previous reports. Patients older than 60 years would likely have a worse survival outcomes than younger patients. The CSS in patients with low tumor grade would be longer than in those with high tumor grade. CEA levels positive is associated with worse prognosis and the prognosis of patients with lymphatic metastasis might be worse than those without. Currently, there exists controversy regarding the long-term benefit of primary tumor resection in stage IV and many prior studies suggest there is no benefit to survival with primary tumor resection^15, 16^, while others suggest a clinical benefit with surgical resection^17-19^. In our study, we found the patients could significantly be beneficial from primary tumor resection with remarkable improvement of CSS. That might be because removal of primary tumor could prevent future tumor related complications including bleeding, obstruction and perforation, thereby avoiding the decreased quality and survival of patients' life. Notably, the chemotherapy also was a striking prognostic factor for BM that had been demonstrated in many reports^12, 20, 21^. Points for chemotherapy extended across the full range of point axis, representing much more points than any other variables, which demonstrated the relationship between normalized chemotherapy and better survival.

For example, a 70-year-old (25 points) synchronous BM patients from CRC with lymphatic metastasis (40 points), grade II (0 points), CEA positive (35 points) who undertake primary cancer surgery (0 points) and chemotherapy (0 points) has a total of 100 points, resulting the estimated 1-, 2-, 3-year CSS of 58.0%, 32.0% and 18.3%, compared to the total of 250 points with estimated 1-year CSS of less than 10% if the same patients don't receive neither surgery or chemotherapy. The 1-, 2- and 3-year CSS of patients with chemotherapy were both remarkably improved combined with surgical resection of primary tumor, which could further guide the treatment of CRC patients with synchronous BM.

A Turkish research^22^ in 2015 demonstrated that palliative radiotherapy (p=0.001, HR 0.51) was an independent prognostic factors for survival of patients with BM,. Other researches have also proved radiotherapy could improve the patients' prognosis ^12, 23^. However, our nomogram showed an interesting result: The radiotherapy failed to be one of independent prognostic factors. So we held the view that radiotherapy had better efficacy in relieving bone pain rather than in treating BM, and there was no significant difference in CSS between patients with and without radiotherapy.

Our finding showed that chemotherapy had a striking impact on the prognosis and could significantly improve CSS of patients. However, it was unclear about the relevant detailed information for chemotherapy regimen and cycle in the SEER database. In addition, because the SEER database only represents the population in the US, which lacks data on Asian population or other races, leading to the limitation in its widespread use.

## Conclusions

Although the patients with synchronous BM only accounted for a small proportion in CRC patients, the incidence has increased in recent years with poor prognosis. Accurate assessment of prognosis and survival at the diagnose of CRC with synchronous BM would be beneficial for patients. Here, the nomogram based on readily available clinicopathologic factors presented high accuracy to precisely estimate the individualized CSS, which could further provide better decision making to both physicians and patients.

## Figures and Tables

**Table 1:** Clinical and tumor characteristics in colorectal cancer patients with synchronous bone metastasis.

Characteristics	Patient with BM (N=342)	%	Univariable Analysis P
Age, years			.001
<60	147	43.0	
≥60	195	57.0	
Gender			.802
Male	202	59.1	
Female	140	40.9	
Primary tumor site			.066
Ascending colon	146	42.7	
Descending colon	81	23.7	
Rectum	115	33.6	
Tumor grade			.000
Grade I, II	216	63.1	
Grade III, IV	126	36.9	
Primary tumor size, cm			.203
<3	36	10.5	
3≤ <5	106	31.0	
≥5	200	58.5	
Pathological type			.008
Adenocarcinoma	290	84.8	
Mucinous adenocarcinoma	25	7.3	
Signet-ring cell carcinoma	13	3.8	
Other	14	4.1	
CEA			.153
-	64	18.7	
+	278	81.3	
AJCC T stage			.289
T1, T2	50	14.6	
T3, T4	292	85.4	
AJCC N stage			.159
N0	78	22.8	
N1, N2	264	77.2	
Extra-osseous metastasis			.392
No	74	21.6	
Yes	268	78.4	
Primary tumor resection			.004
No	112	32.7	
Yes	230	67.3	
Radiotherapy			.150
No	226	66.1	
Yes	116	33.9	
Chemotherapy			.000
No	116	33.9	
Yes	226	66.1	

BM: bone metastasis; N: number; CEA: carcinoembryonic antigen

**Table 2:** Multivariable Cox model of CSS after forward selection of variables with P<0.20 in univariate Kaplan-Meier analyses.

Characteristics	HR	CI	P
Age, years			
≥60 vs. <60	1.398	1.081-1.809	.011
Tumor grade			
Grade III, IV vs. Grade I, II	1.623	1.246-2.113	.000
CEA			
+ vs. -	1.626	1.158-2.282	.005
AJCC N stage			
N+ vs. N0	1.730	1.250-2.394	.001
Primary tumor resection			
No vs. Yes	2.022	1.531-2.669	.000
Chemotherapy			
No vs. Yes	3.920	2.947-5.215	.000

HR: hazard rate; CI: confidence interval
